# The Role of Inflammatory Hematological Markers in Predicting IVF Success

**DOI:** 10.5935/1518-0557.20200050

**Published:** 2021

**Authors:** A Seval Ozgu-Erdinc, Bugra Coskun, Ayçağ Yorganci, Necati Hancerliogullari, Nafiye Yilmaz, Yaprak Engin-Ustun

**Affiliations:** 1 Ministry of Health Ankara City Hospital, Ankara, Turkey; 2 Ankara Liv Hospital, Ankara, Turkey; 3 Etlik Zübeyde Hanim Women's Health Education and Research Hospital, Ankara, Turkey

**Keywords:** infertility, in vitro fertilization, inflammatory hematologic markers, platelet/lymphocyte ratio

## Abstract

**Objective:**

To investigate the predictive role of inflammatory hematological markers on treatment success in in vitro fertilization (IVF) patients.

**Methods:**

In this study, we analyzed the data from the patients who admitted to our IVF center, and we recorded demographic characteristics, medical histories, laboratory biomarkers, cycle characteristics, and IVF outcomes from the patients’ files. We assessed the value of white blood cell (WBC) counts, neutrophil/lymphocyte ratio (NLR), monocyte/lymphocyte ratio (MLR), platelet/lymphocyte (PLR), mean platelet volume (MPV) and platelet distribution width (PDW) of the patients from their complete blood count. We compared these values in terms of predicting positive HCG test after embryo transfer (ET).

**Results:**

There were 132 patients, of which 63 (47.7%) were treated for male factor, 43 (32.6%) for unexplained infertility, 19 (14.4%) for diminished ovarian reserve, 5 (3.8%) for endometriosis and 2 (1.5%) for hypogonadotropic hypogonadism. After ovarian stimulation and oocyte retrieval, 115 patients underwent embryo transfer, and 28 patients had a positive HCG test (24.3%). The positive HCG group had a statistically lower PLR when compared to the HCG (-) group (*p*=0.02). In the ROC analysis, PLR was significant in predicting positive HCG (*p*=0.028). However, when we added other factors to the model, only age and MII oocyte count were successful in predicting pregnancy outcomes in a logistic regression analysis.

**Conclusion:**

According our results, inflammatory hematological markers were not effective in predicting IVF success.

## INTRODUCTION

In vitro fertilization (IVF) is an effective and successful medical treatment option for couples when other infertility treatments fail to achieve pregnancy. The female factor or male factor infertility is among the indications for IVF in the presence of a detectable problem. However, approximately one-third of the couples are classified as having unexplained infertility after various attempts to establish the underlying cause ^(Vitek et al., 2013)^.

Many factors affect IVF success. One of the most important factors is implantation failure. For the implantation to be successful there is a need for a healthy embryo, a receptive endometrium, and maternal cellular/humoral tolerance. If there are problems with these, the implantation might fail ^(Ozkan et al., 2014)^. The receptive endometrium exhibits signs of inflammation with various cytokines secreted from hematopoietic cells ^(Dekel et al., 2010)^. Successful implantation and placentation require well-balanced inflammation and immune tolerance. Apart from white blood cells, the role of platelets in the release of mediators that cause local changes in the inflammatory process is very relevant. The hematological marker that shows the best platelets function is the mean platelet volume (MPV). Large platelets are more effective in terms of enzymatic activities ^(Mangalpally et al., 2010)^. Platelet distribution width (PDW) is a reflection of platelet heterogeneity ^(Vagdatli et al., 2010)^. Neutrophil to lymphocyte ratio (NLR), monocyte to lymphocyte ratio (MLR), and platelet to lymphocyte ratio (PLR) are other hematological markers that show systemic inflammatory status ^(Afari & Bhat, 2016^; ^Hwang et al., 2017^; ^Wang et al., 2018^; ^Nishijima et al., 2015)^.

The systemic inflammatory response could be easily assessed from a whole blood analysis by calculating NLR, MLR, PLR, and evaluating MPV and PDW. In this retrospective study, we aimed to investigate the role of systemic inflammatory hematological markers in predicting IVF success.

## MATERIALS AND METHODS

The Institutional Review Board of the University of Health Sciences Ankara Dr. Zekai Tahir Burak Women’s Health Education and Research Hospital (02.06.2014-22) approved the study. For this retrospective cohort study, we recruited 132 patients from our hospital’s IVF center files, between June 2014 - June 2015. We obtained the following data from the patients’ files, age, body mass index (BMI), medical and infertility history, thyroid-stimulating hormone (TSH) and prolactin (PRL) levels, day 3 hormone levels of follicle-stimulating hormone (FSH), luteinizing hormone (LH), estradiol (E2) and progesterone (P), and ultrasonographic features. We excluded patients with tubal-factor, severe endometriosis, endometrioma ≥ 5cm, systemic or autoimmune diseases, continuous use of glucocorticoid or anti-inflammatory drugs, who had signs of acute infectious disease, endocrinopathy and hematologic diseases. Additionally, at our center, all the patients routinely underwent psychiatric evaluation before the IVF therapy and they received psychological support throughout the cycle. Thus, we excluded stress that could affect the complete blood count (CBC).

From the patients’ records, we noted ovarian stimulation protocols, gonadotropin type, total gonadotropin dose, human chorionic gonadotropin (HCG) type, the total number of oocytes retrieved, metaphase II (MII) oocyte count, fertilization rate, and the number of embryos. We calculated the fertilization rate (FR) as the percentage of fertilized oocytes to MII oocytes. A positive HCG test was the primary endpoint, which was achieved 14 days after ET. We defined clinical pregnancy as the presence of an intrauterine gestational sac seen on transvaginal ultrasound.

We recorded the CBCs performed before controlled ovarian stimulation in the routine evaluation tests. We ran the CBC test using the Siemens Healthcare Diagnostic Item ADVIA 2120i device. From the CBC test, we analyzed white blood cell count, NLR, MLR, PLR, MPV, and PDW for all the patients.

### Statistical analysis

We transferred the data to a computer using the Statistical Package for the Social Sciences version 23.0 (SPSS, Chicago, IL). We then assessed data distribution using the Kolmogorov-Smirnov test. We presented continuous and normally distributed variables as means ± standard deviation, and we investigated intergroup differences using the Student’s t-test. We expressed the continuous variables with non-normal distribution as medians (minimum-maximum), and we analyzed the differences between variables using the Mann-Whitney U test. We assessed the differences between categorical data using the chi-square test. In order to examine the success of hematologic markers for the prediction of positive HCG result; we evaluated the receiver-operator curve (ROC) and logistic regression analysis to evaluate the association between dependent and independent variables. A p-value of <0.05 was considered statistically significant.

## RESULTS

One hundred and thirty-two patients met the inclusion and exclusion criteria. Of them, we treated 63 (47.7%) for male factor, 43 (32.6%) for unexplained infertility, 19 (14.4%) for diminished ovarian reserve, 5 (3.8%) for endometriosis and 2 (1.5%) for hypogonadotropic hypogonadism. We suppressed the pituitary using the gonadotropin-releasing hormone (GnRH) antagonist protocol in 80 (60.6%) patients, using the long agonist GnRH therapy in 49 patients (37.1%). There was one natural cycle IVF, and the pituitary was not suppressed in two hypogonadotropic hypogonadism patients. Recombinant FSH, human menopausal gonadotropin (hMG) or both were used for controlled ovarian stimulation. To trigger ovulation, we administered human chorionic gonadotropin (hCG) (118 patients, 89.4%) or recombinant choriogonadotropin alfa (14 patients, 10.6%) when we reached at least two 18 mm dominant follicles. After ovarian stimulation and oocyte retrieval, we could not transfer embryos in 17 patients (12.8%). Of the 115 patients submitted to ET, HCG was positive in in 28 patients (24.3%). There was a negative HCG test result in 87 patients (75.7%). The rate of implantation per ET was 24.3% (28/115), the clinical pregnancy rate was 20.9% (24/115), the pregnancy loss rate was 1.74% (2/115), and the live birth rate was 18.3% (21/115). One of the patients had pregnancy loss after 20 weeks and we detected ectopic pregnancy in 2 patients.

According to their HCG test result, we compared the patients in terms of age, BMI, infertility duration, number of cycles, FSH, LH, E2, P, TSH, and PRL. There was a statistically significant difference in the age of the patients, and the patients who had positive HCG test were younger (*p*=0.002) (Table 1).

**Table 1:** Comparison of intergroup demographic data and baseline hormone values

	HCG (+) (n=28)	HCG (-) (n=87)	*p* value
Age	27 (20-36)	31 (20-43)	0.002
BMI (kg/m^2)^	24.87 (19.13-34.17)	23.87 (17.63-38.14)	0.609
Duration of infertility	4.25 (1-15)	5 (1-21)	0.261
Number of cycles	1 (1-3)	1 (1-3)	0.904
E2	42.37 (11-89.87)	38.31 (11-104)	0.341
FSH	6.32±2	7.15±2.4	0.152
LH	4.89 (0.01-24.9)	4.49 (0.1-15.87)	0.247
TSH	1.95 (0.05-4.8)	1.98 (0.01-4.9)	0.711
PRL	13.58 (5.73-47)	11.77 (2.28-44.2)	0.413
P	0.6 (0.21-1.7)	0.49 (0.13-2.43)	0.427

BMI=Body mass index; E2=Estradiol; FSH=Follicle Stimulating hormone; LH=Luteinizing Hormone; TSH=Thyroid Stimulating Hormone; PRL=Prolactin, P=Progesterone

When we compared the treatment protocol and treatment results between the groups, there were significant differences in the total number of oocytes obtained, number of MII oocytes, fertilization rate and the number of embryos, and they were higher in the HCG positive group (*p*<0.05). There was no significant difference between the groups in terms of gonadotropin type, total gonadotropin dose and endometrial thickness (*p*>0.05) (Table 2).

**Table 2:** Comparison of treatment protocol and treatment results between groups

	HCG (+) (n=28)	HCG (-) (n=87)	*p* value
Gonadotropin Type			0.867
rFSH (n)	23	52	
hMG (n)	2	15	
rFSH+hMG (n)	3	20	
Total gonadotropin dose (IU)	2175 (625-4500)	2280 (1025-5400)	0.65
Endometrial thickness (mm)	10.4 (7.5-15)	10 (6.5-15)	0.794
Total number of oocytes (n)	13 (3-28)	9 (1-24)	0.001
Metaphase II oocyte count (n)	10.5 (2-25)	6 (0-21)	<0.001
Fertilization rate (%)	63.96 (11.11-100)	60 (0-100)	0.025
Number of embryos (n)	7 (1-19)	5 (0-20)	<0.001

rFSH=Recombinant Follicle Stimulating Hormone; HMG=Human Menopausal Gonadotropin

**Figure 1:**
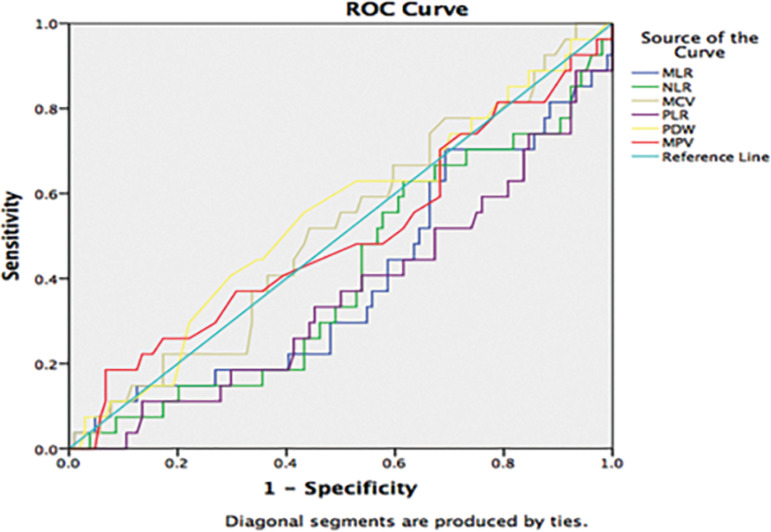
Receiver-operator curve analysis of preoperative hematologic inflammatory markers for the prediction of positive HCG test

We compared the groups in terms of WBC, NLR, MLR, PLR, MPV, and PDW. There was a statistically significant difference only in PLR, and it was lower in the group with a positive HCG result (*p*=0.02) (Table 3). In the ROC analysis, PLR was significant in predicting pregnancy (*p*=0.048) (Figure 1). However, in the logistic regression analysis, other factors (age, BMI, basal hormones, MII oocyte count) were added to the model and as a result, only age and MII oocyte count were successful in predicting pregnancy outcomes.

**Table 3:** Intergroup inflammatory hematologic markers comparison

	HCG (+) (n=28)	HCG (-) (n=87)	*p* value
WBC (min.-max.)	6610 (4680-11000)	7000 (4160-16710)	0.816
NLR (min.-max.)	2 (0.68-4.84)	2.17 (0.74-10.01)	0.344
MLR (min.-max.)	0.18 (0.09-0.65)	0.2 (0.1-0.82)	0.068
PLR (min.-max.)	12167 (5455-21146)	13814 (5814-37358)	0.02
MPV (fL) (min.-max.)	9.8 (0.15-12.2)	9.95 (0.27-14.1)	0.671
PDW (fL) (min.-max.)	15.8 (10.7-16.9)	15.6 (10.1-17.7)	0.570

WBC=White blood cell; NLR=neutrophil/lymphocyte ratio; MLR=monocyte/lymphocyte ratio; PLR=platelet/lymphocyte ratio; MPV=mean platelet volume; PDW=platelet distribution width

## DISCUSSION

Pregnancy begins and continues as a result of an embryo implantation in the endometrium. During the implantation window, the inflammatory cells are active in the endometrium, including natural killer cells, macrophages, and dendritic cells, but not the neutrophils ^(Chavan et al., 2017^; ^Seçkin et al., 2016)^. Despite the abundance of inflammatory cells, maternal immune tolerance should develop towards the semi allogeneic embryo for successful implantation ^(Song & Shi, 2014^; ^Yıldız et al., 2016)^. Any disintegration and imbalance between these immune responses might result in implantation failure or early pregnancy loss, and patients might face infertility problems. In particular, inflammatory cells and cytokines secreted from these cells are elevated in unexplained infertility cases (An et al., 2015; ^Ozkan et al., 2014)^. Inflammation caused by organic diseases, such as endometriosis, polycystic ovary syndrome (PCOS) or obesity, is a well-known cause of infertility ^(Cho et al., 2008^; ^Çakıroğlu et al., 2016)^. However, ^Sjaarda et al. (2018)^ tested the inflammatory status by high-sensitive C-reactive protein (hs-CRP) and found that low-grade inflammation is very common (20-40%) in reproductive-age women, including normal-weight women. Apart from hs-CRP, systemic inflammatory status could be assessed by NLR, MLR, and PLR ^(Mertoglu et al., 2019^; ^Madendag et al., 2018)^. On the other hand, platelets release a high rate of inflammatory mediators to the environment during inflammation ^(Thomas & Storey, 2015)^. The hematological marker that best demonstrates the function of platelets is MPV ^(Yılmaz et al., 2017)^. During low-grade inflammation as the platelet size increases, more enzymatic activity and a greater number of cytokines are released into the environment ^(Mangalpally et al., 2010^; ^Gasparyan et al., 2011)^. PDW is a more specific indicator of platelet activation than MPV, but it would be more useful to evaluate MPV and PDW together in predicting platelet activation ^(Vagdatli et al., 2010)^. In our study, we investigated the role of inflammatory hematological markers in predicting IVF success. Although PLR was statistically lower in HCG-positive patients, only age and MII oocyte count was significant to predict HCG positivity in logistic regression analysis. The small number of patients might have caused the PLR to be insignificant.

There are few studies investigating the role of inflammatory hematological markers in predicting IVF success. ^Çakıroğlu et al. (2016)^ investigated the role of hematological markers in infertile patients with PCOS in predicting the relationship between obesity and IVF success. As a result, they found that MPV values were negatively correlated with clinical pregnancy, and implantation rates in IVF patients with PCOS and PLR value were positively correlated with pregnancy loss. In a more recent study, there was a positive correlation between lymphocyte count and FR, and a negative correlation between PLR and implantation among unexplained infertility patients. Thus, high PLR (elevated platelets and/or decreased lymphocytes) were associated with high pregnancy loss or fewer implantation rates. We also found decreased PLR ratio in HCG positive patients. The receptivity of the endometrium has recently been increasingly emphasized in patients with unexplained infertility. In fact, it is seen as the main cause of implantation failure and early pregnancy loss in patients who underwent IVF for unexplained infertility ^(Jasper et al., 2006)^. Inflammatory cells increase the number of cytokines accumulating in the endometrium during the implantation window. Subsequently, implantation happens by the invasion of the endometrium with tissue remodeling and angiogenesis. Any unbalance between these processes could lead to implantation failure. Considering both the results of the abovementioned studies and the active role of platelets in hemostasis and inflammation, PLR might be a novel marker for assessing implantation success. This hypothesis should be evaluated by large prospective studies.

A preliminary study, assessed levels of lymphocyte subpopulations and cytokines in patients with unexplained infertility during the days of the implantation window ^(Fornari et al., 2002)^. Ovarian stimulation increased the number of leukocytes and lymphocytes, and restored some of the immune alterations found among infertile patients. Therefore, ovarian stimulation might alter the systemic inflammatory hematologic parameters. All the studies about this topic, including our study, used baseline hematologic parameters prior to ovarian stimulation protocol onset. Considering that ovarian stimulation changes the number of leukocytes and lymphocytes, whole blood analysis performed on different days during an IVF cycle might yield results that are more interesting.

Our study has many limitations. First, it is a retrospective study, which makes it susceptible to selection biases. Secondly, it consists of relatively small numbers. Although we found decreased levels of PLR in HCG positive patients, only age and MII oocyte were significant for predicting IVF success in logistic regression analyses. These results are in accordance with other analyses published in the literature ^(van Loendersloot et al., 2010^; ^Elizur et al., 2005)^. On the other hand, the study participants consisted of a heterogeneous group. However, 80% of the study patients (male factor and unexplained infertility) were normal ovulatory patients.

In conclusion, our results showed that inflammatory hematological markers (WBC, NLR, MLR, PLR, MPV, and PDW) did not play a significant role in predicting IVF success. However, among these parameters PLR seems to be a more promising marker than others are, suggesting that larger prospective studies might confirm its value in the future.
